# Does “distance lend enchantment”? Public attitudes to deepfake technology in the United States

**DOI:** 10.1177/09636625251374850

**Published:** 2025-10-26

**Authors:** Elena Denia, John Durant

**Affiliations:** Australian National University (ANU), Australia; Universitat Politècnica de València (UPV), Spain; Massachusetts Institute of Technology (MIT), USA

**Keywords:** artificial intelligence, deepfake, public attitudes, public engagement

## Abstract

We describe a focus group study of public perceptions of “deepfake” technology, that is, digitally manipulated videos that show people saying or doing things they never really did. The study was designed to explore the relationship between degree of closeness to or familiarity with technology and attitudes toward it. We find that in this case, publics that are closer have more positive and nuanced attitudes. In such cases, at least, it appears that distance does not lend enchantment. We suggest why this may be the case and propose further related research designed to test the conclusions reached here.

## 1. Public attitudes toward science and technology

Sociological research on public attitudes toward science and technology suggests, at the very least, that the situation is highly complex. Most national studies that have tried to measure attitudes to science and technology in general (“general attitudes”) have found these to be fairly positive, and there is long-term trend data for the United States that shows stability in such measures over decades. But this apparent simplicity is misleading. For example, even the most apparently stable general attitudes can change as cultures change. Also, it has been known for some time that general attitudes are poorly related to specific attitudes on particular scientific and technological topics. While on some specific issues better informed respondents tend to have more positive attitudes, on others they tend to be more critical. Again, in recent decades, political partisanship has greatly affected different publics’ views of science in the United States. And the situation gets only more complicated when we analyze aggregate attitudinal measures, asking (for example) about the relationship between attitudes and other variables, such as interest in or knowledge of science and technology (Allum et al., 2008; Besley and Hill, 2020; Durant, 2022; Durant and Evans, 1995; Funk, 2020), or about the related concepts of trust in scientists and scientific institutions (Achterberg et al., 2015; Dixson et al, 2022).

On the question of the relationship between attitudes and knowledge, we are faced with two contrasting and seemingly contradictory views. First, there is the familiar view that, “to know science is to love it” (Turney, 1998); that is, the closer to or more familiar with science people are, the more positive will be their attitude toward it. It has been argued that such a view underlies a great deal of practical activity aimed at improving public understanding of science, and the presence of this view among practitioners has been detected in a number of empirical studies (e.g. Lewenstein, 1992). Over against this point of view, however, is a quite different notion coming from sociology of science. On this second view, something close to the opposite idea has been proposed. Here are two sociologists of science setting out their case (Collins and Pinch, 2014: 3):

Another idea introduced in this volume is the notion that in science and technology, as in love, “distance lends enchantment.” That is to say, scientific and technological debates seem to be much more simple and straightforward when viewed from a distance. When we find ourselves separated from our loved-ones we remember only why we love them; the faults are forgotten. In the same way, science and technology, when understood through others’ inevitably simplified accounts, look artless. Closer to the centre of a heated debate, the less pre-determined and more artful do science and technology appear. The irony is that, quite contrary to what common-sense might lead one to expect, it is often that the greater one’s direct experience of a case, the less sure one is about what is right.

Collins and Pinch are offering their view, not as a contribution to attitudinal studies per se, but rather as a claim about the ways in which certainty is established among groups of expert scientists and technologists. This claim has been much developed and modified by other sociologists. For example, in his much admired analysis of the accuracy of strategic ballistic missile guidance system, MacKenzie (1993) has argued for the existence of a “Certainty Trough,” in which expert assessments of the uncertainty attaching to estimates of the accuracy of strategic ballistic missile guidance systems were higher among expert developers of guidance technology and among critical experts who favored alternative approaches, but lower among potential consumers of this technology—those who, as he put it, “believe what the brochures tell them.” In her study of climate modelers, however, Lahsen (2005) qualified this conclusion by questioning that knowledge producers are always the best judges of the accuracy of their own models.

In the present study we are not concerned, as MacKenzie was, with expert perceptions; rather, ours is a study of different publics, none of whom are technological experts. Also, we are not concerned with issues of “certainty” and “uncertainty,” but instead with attitudinal perceptions. None of the participants in our study seriously questioned the certainty or reliability of the technology under consideration. However, they were variously concerned about its personal, social, and even political implications. Taking into account the more general connotations of the term “enchantment,” we ask here: is greater closeness to or familiarity with a specific technology associated with more positive attitudes, or more negative ones? Is it indeed the case that “to know science is to love it,” or rather is it the case that, in this attitudinal sense, “distance lends enchantment”?

Our study was designed to explore this question empirically. We have chosen one specific area of science and technology, namely recent developments in Artificial Intelligence (AI) that are associated with so-called deepfake technology (see Section 2). We have been enabled to explore the relationship between “closeness” to this topic and feelings about it by taking advantage of the fact that the MIT Museum—where we were both located at the time this research was undertaken—offered highly relevant exhibits and public programs in the period 2022–2023. These resources allowed us to convene three focus groups (FGs) comprising individuals with very different degrees of “closeness” to or familiarity with deepfake technology. While the results reported here should not be generalized uncritically to other areas and issues, they do provide in-principle testable insights into the relationship between public knowledge of and attitudes toward technology.

## 2. Deepfake

The manipulation of still and moving images has a history almost as long as the histories of photography and film-making. However, in recent decades, AI has been widely exploited in the increasingly sophisticated manipulation of visual and audio content in ways that are potentially deceptive and often—though not always—controversial.^1^ The term “deepfake” appears to have originated in 2017 from a Reddit user named “deepfakes” (Cole, 2018). From around this time, videos started to appear online in which politicians’ and celebrities’ faces were superimposed on the bodies of other people (including porn stars), and these and other applications provoked an increasing amount of critical attention. As one commentator has observed (Wong, 2022),

It was 2018, and the world as we knew it—or rather, *how* we knew it—teetered on a precipice. Against a rising drone of misinformation, *The New York Times*, the BBC, *Good Morning America*, and just about everyone else sounded the alarm over a new strain of fake but highly realistic videos. Using artificial intelligence, bad actors could manipulate someone’s voice and face in recorded footage almost like a virtual puppet and pass the product off as real. In a famous example engineered by *BuzzFeed*, Barack Obama seemed to say, “President Trump is a total and complete dipshit.”

Deepfake is not the only application area that has recently focused public attention on the wider ethical and social implications of AI. In particular, large language model-based chatbots such as the Generative Pre-trained Transformer (GPT) series have attracted enormous public interest, especially since the launch of ChatGPT in November 2022. In December 2022, tech entrepreneur Elon Musk announced that AI was at risk of being “woke”; in January 2023, the Microsoft Corporation announced an investment of over $10bn in OpenAI; in February 2023, New York Times columnist Kevin Roose published an extraordinary transcript of his own conversation with Bing AI, in the course of which the AI was reported to have assured him, “I Want to Be Alive.”; in March 2023, Musk and tech entrepreneur Steve Wozniak co-signed a call for a 6-month moratorium on the training of AI systems more powerful that GPT-4; and in May 2023 AI pioneer Geoffrey Hinton resigned from his position at Google in order to be able speak freely about what he saw as the dangers of a technology in whose development he had played a major part. By the time that the US Congress came to conduct its first hearing on oversight of AI, also in May 2023, ChatGPT had become one of the most widely talked-about technologies of recent times (Bartholomew and Mehta, 2023).

The MIT Museum contained some exhibits intended to engage audiences with some of these developments. An exhibition entitled *AI: Mind the Gap* announced that it “compels us to question [AI’s] potential and recognize its risks.” This exhibition included an exhibit on generative AI called *Let’s Write a Poem Together*, which allowed visitors to create a poem collaboratively with GPT-3 (at that time ChatGPT had not yet been released), and another on deepfake, called *True or False. True or False* had two sections: an art installation, *In Event of Moon Disaster;* and an interactive video-wall, *Detect a Fake. In Event of Moon Disaster* was a museum implementation of an immersive multi-platform art work using deepfake technology to produce media that would have existed in the event that the 1969 Apollo 11 mission had failed (Panetta and Burgund, 2019). *Detect a Fake* was an array of videos of people talking, in which visitors were invited to guess which videos were real and which were fake.

*True or False* was the immediate inspiration for the research reported here. In fall 2022, we co-taught an MIT undergraduate class (STS.036: “Science in American Life”), in the course of which our students undertook a pilot evaluation of this exhibit. In February 2023, the MIT Museum offered a 3-session evening class, entitled “Make a Fake,” in which MIT Media Lab graduate student Matt Groh—himself a co-author of *Detect a Fake*—taught a group of adult students the basic principles of generative AI, guiding them in both how to make and how to detect simpler deepfakes. Following the final session of this class, all of the students participated in a moderated discussion of deepfake, chaired by one of us. This recorded conversation became the first Focus Group (“FG#1”) of the present study. It was then followed up by two further FGs, one involving participants recruited from among general visitors to the MIT Museum, and another recruited from members of the general public visiting a large shopping mall in downtown Boston. The rationale behind these choices is outlined in Section 3.

## 3. Methodology

For the present study we organized three FGs, each designed to bring together groups calculated to be at different social distances from deepfake technology.^2^ FG#1 comprised the students in the short course *Make a Fake*, which was held in the MIT Museum on 13th, 20th, and 27th February 2023. We term the graduates of this short course a group of the “engaged public” for deepfake technology. FG#2 comprised recruits from among the general visitors to the MIT Museum. We have known for some time that on average such visitors tend to be highly interested in and attuned to science and technology, and so we term this a group of the “interested public.” Finally, FG#3 comprised recruits from among general visitors to downtown Boston, many of whom were shoppers visiting the Prudential Center Mall. We term this a group of the “general public.”

There are several limitations to this study. The sample size (3 FGs) is admittedly small, though we believe it is justified by the ability to perform a comparative analysis of three significantly different publics. Again, the study is purely qualitative—no quantitative estimates can be obtained from it. This is partly because of the small sample size, and partly because we have chosen qualitative methods for the analysis. We agree with Lunt and Livingstone that the FG is a valid method of social research in its own right, and not (as Merton and others argued) a mere adjunct to other, primarily quantitative methods. Furthermore, we agree with these authors that the FG should be regarded as, “a simulation of various aspects of social communication” (Lunt and Livingstone, 1996). We see the FG as an admittedly contrived conversation that can reveal much about the ways in which particular social groups deliberate about particular topics. Finally, we should note that the three publics sampled here are not even approximately of the same size. Those who are “engaged” (FG#1) or merely “interested” (FG#2) in science are minorities; most members of the public would fall into our “general” (FG#3) category. This should be borne in mind in judging the significance of our findings.

In summary, the FGs were recruited as follows:

FG#1 (engaged public): 12 participants in a 3-session *Make a Fake* class; the students were offered a full course refund in return for their participation in the study, which took place immediately following the final session of the class on Monday 27 February 2023.
FG#2 (interested public) 14 visitors to the MIT Museum who volunteered to participate in the discussion after being recruited in the museum itself. The activity was scheduled for the week following recruitment, Monday 24 April 2023, and as compensation for their involvement participants were offered a refund of their museum admission fee, and two more tickets for another day.
FG#3 (general public): 13 pedestrians who were recruited with the help of 4 MIT volunteers working in the immediate environs of the Boston Public Library and the adjacent Prudential Center Shopping Mall. Participants were offered a $70 Amazon Gift Card in consideration for 1 hour of conversation on the same day of their recruitment, Saturday 27 May 2023.

The self-stated personal and/or professional backgrounds of FG participants are listed in Supplemental Appendix A. As will be seen, the composition of the three FGs was markedly different: many members of FG#1 had clear professional reasons for wanting to know more about how to make deepfakes; many members of FG#2 had substantial educational experience and/or expertise in science and technology; and most members of FG#3 had no special relationship to deepfake technology in particular, or science and technology in general. None of the participants in FGs #2 and #3 knew about the topic of the conversation in advance. Care was taken to ensure that in each case the FG samples were diverse in gender and age (all participants were over 21 years of age).

Each FG conversation lasted 1 hour, and each was recorded on different devices in order to capture all voices across the rooms. The data were anonymized and transcribed under the review of both authors. The final transcripts of the three conversations can be consulted in the open access EU repository *Zenodo* (Denia and Durant, 2024), operated by the European Organization for Nuclear Research (CERN). A standard question protocol was followed in each group, with modifications only where these were required by the immediate context of recruitment (e.g. we began FG#1 by asking participants about their experience in the *Make a Fake* course). The FG Protocols are provided in Supplemental Appendix B. In the cases of FGs #2 and #3 only, a brief and neutral definition of deepfake technology was provided: “Deepfakes are digitally manipulated videos that show people saying or doing things they never really did.”

The transcripts were analyzed qualitatively, with a view to identifying the distinctive analytical categories (point of view, attitude, tone, media associations, and frames; see Section 2) present in each conversation. No attempt was made to quantify the results in a coding frame, as the sample size was considered too small to make this worthwhile. Instead, each author undertook close readings of the transcripts independently in a search for characteristic modes of discourse; and the results were then shared between us and found to be broadly similar. This resulted in the identification of a number of analytical categories that together served to highlight the most striking features of each conversation. In the results section, each distinctive analytical category is exemplified with multiple quotations from the relevant transcripts. Individual FG participants are referred to for purposes of quotation using the following convention: number of FG (1, 2, or 3), followed by participant number (see Supplemental Appendix A). Thus, for example, the third participant in FG#2 would be referred to as: P2.3, and so on.

## 4. Results

The three FGs succeeded in capturing three coherent conversations about deepfake technology. Judging from the transcripts, at the time that we conducted this research AI was very present in the US public mind. This being said, we wish to capture the extent to which these three conversations were both similar and different. By similarity, we have in mind for example that all three groups:

1. Displayed a general awareness of AI, with ChatGPT being the single most salient example (see the comments about the prominence of media coverage of this technology at the time of our Focus Groups, in Section 2);
2. Displayed attitudes toward AI that could broadly be described as ambivalent (i.e. recognizing both potential benefits and potential disbenefits);
3. Were aware, at least to some degree, of the problem of “algorithmic bias” in the operation of these technologies.

None of our FGs bought into the idea that deepfake technology was either wholly good or wholly bad.

On item 1, general awareness of AI: P1.5 said of ChatGPT that it “Sounds like a high school senior or a very beginning college level”; P2.5 said, “we can see that the ChatGPT can now answer all of our questions”; and P3.8 said, “ChatGPT. It’s everywhere today.” On item 2, the display of ambivalent attitudes: P1.9 said, “I can [detect a deepfake] until I can’t … and that’s the part that’s a little concerning. I mean, right now I think I do, but I don’t think that’s going to stay that way”; P2.9 said,

we can try to use it [AI] in healthcare, we can try to use it in finance and this and that, but we have this tool and it’s exciting and it’s got so many users, but it wasn’t driven by a problem looking for a solution. It was driven by “we have this technology now and we’re able to utilize this technology to predict things. So let’s try to figure out what we can do with it.”

On item 3, algorithmic bias, P1.12 said, “When we teach kids to look for bias, we usually focus on articles, but this opens a whole new venue [sic]”; P2.4 said, “.. .they [people] don’t realize that the data is trained on biased things”; and P3.10 said, “… humans might impose their bias on the AI system.”

So much for similarity between the focus groups. Table 1 captures some of the key differences between the three conversations.

**Table 1.** Differences between the three focus group conversations.

Analytical category	Engaged public FG#1	Interested public FG#2	General public FG#3
(i) Point of view	1st person (“Me”/“We”/“Us”)	3rd person (“They,” as in the sense of other less well-informed people)	3rd person (“They,” as in the sense of the authors and operators of AI)
(ii) Attitude	Generally positive	Generally ambivalent	Generally negative
(iii) Critical Evaluation and Tone	Highly nuanced: the vast majority express both positives and negatives, using emotionally restrained and moderately technical language	Moderately nuanced: half express a weighted opinion, in language that is less restrained, moderately emotional and quite technical	Not nuanced: the vast majority express purely negative views and a few purely positive ones, in language that is relatively unrestrained, often quite emotional and not very technical
(iv) Cultural/Media associations	Lower presence of cultural and mass media images	Moderate presence of cultural and mass media images	High presence of cultural and mass media images
(v) Frames	Social Progress	Deficit Model	Pandora’s Box

By “Point of View,” we mean the preferred pronouns (first person, second person, third person) used in FG conversations, as markers of overall stance of FG participants toward AI/deepfake. By “Attitude,” we mean the general way of thinking or feeling about AI that was expressed by the FGs. By “Critical Evaluation,” we mean the extent to which the FGs revealed a willingness on the part of participants to evaluate multiple perspectives on AI, and by “Tone” we mean the form of conversation characteristic of each FG—more or less restrained, more or less emotional, more or less technical, and so on. By “Cultural/Media Associations,” we mean the extent to which FGs reached for examples of AI from wider cultural sources (mass media, social media, movies, etc.). Finally, by “Frames” we mean prevailing perspectives that serve to identify what really matters in ways that resonate with core values and assumptions. Framing theory was originally developed in the social sciences (e.g. Entman, 1993; Scheufele, 1999), but it has been applied with some success to science and technology (e.g. Nisbet, 2009; Nisbet and Mooney, 2007). In what follows, we present results from the FGs under each of these analytical categories.

### Point of view

Differences in the use of preferred pronouns are some of the most striking contrasts between the three FG conversations. In FG#1, the preferred pronouns are first person (I/me/us), while in both FG#2 and FG#3 the preferred pronouns are third person (they/them). However, third person pronouns are generally used quite differently by FG#2 and FG#3. When FG#2 participants refer to others in the third person, mostly the people they are referring to are other members of the general public (sometimes referred to simply as “other people”) who are presumed to know less about AI than the participants themselves, whereas when FG#3 participants refer to others in the third person, more often than not they are referring to the (sometimes unspecified and by implication unknown) authors or users of AI systems—that is, people far more knowledgeable and powerful in the AI world than themselves. Closely linked to the use of preferred pronouns is the general orientation of FG participants toward our topic. FG#1 participants emphasized the personal utility of AI/deepfake, together with the need to “keep up” with new developments in these fields. FG#2 participants emphasized other people’s deficiencies in understanding and being able to cope with AI/deepfake. Finally, FG#3 participants emphasized the malevolent (mis)uses to which both specified and unspecified users might put AI/ deepfake.

Among the “Engaged Public” (FG#1), the conversation unfolds in the first person and revolves around the self (P1.10: “I’d like to look at anything that have [sic] to do with technology that will expand my mind”). Aligned with this, individuals’ statements emphasize the personal utility of AI, under the general idea of “what this technology can do for me,” and they express concern about keeping up with new technological developments. Participants declare their intentions to make further use of deepfake, for both personal and professional purposes, not only because it can simplify tasks at work but also because it is a source of wonder and fun with high creative potential that offers ways to better understand social reality. Here is a selection of typical quotations: “I’ve been interested in ways in which I work with the machine, and this kind of [AI] synthetic media has been like another avenue that I wanted to explore” (P1.4); “I am trying to build up a portfolio for communication media samples that I’ve produced, so rather than having to put my makeup on, and look pretty, and put the lights … I’m gonna use my [deepfake] clone to do the narration [Laughs]” (P1.6); “I wanted to be able to get some information about this [AI/deepfake] to have good conversations with my students” (P1.12); “We are decorating our home and then there’s this wall that’s empty and we’re thinking about what to put there. And then I’m thinking I could just generate these … like Polaroid pictures of the way we think the house is gonna look in the future” (P.1.7).

Among the “Interested Public” (FG#2), the prevailing point of view is third person. This is because a central element of the conversation is a contrast between FG participants, who see themselves as better informed, and the majority of the public, who are viewed as being not so well-informed or discriminating with respect to AI/deepfake (P2.11: “Now is the time to be making sure that people know the critical thinking”). To be noted here is the fact that virtually the entire group has a technoscientific background. Among this group, the prevailing point of view is one in which other people’s deficiencies and unreliabilities are key challenges to be faced in the social uptake of the technology. Again, here are some illustrative quotations: “I’m super concerned about public perception of what they think AI is” (P2.12); “The trouble is that some people are not bright enough to realize this [is a deepfake]” (P2.4); “I think society isn’t ready for it because it’s not willing to consider the amount of information that has to go into making those things” (P2.3); “people already don’t check sources of things they believe, whatever they read on the internet, they go to the same source for their information and don’t realize that it’s false. And I think this is just going to mushroom …” (P2.13); “we know all you kids are gonna be on TikTok; here’s what you do to make it safe” (P2.11).

Among the “General Public” (FG#3), the narrative again relies on the third person, but now the focus of interest is very different. Rather than a supposedly less-well-informed general public, the people who are commonly invoked by the use of the impersonal third person “they” are a variety of (often ill-defined) sources of deepfakes—sometimes scientists or engineers, sometimes corporations, sometimes governments, and sometimes scammers. These third person sources have in common that they are at best dimly seen and generally distrusted. “They” are responsible for most of the abuses and misuses that are taking place, as a new technology is applied in service of “their” more or less hidden agendas. Here are some illustrative quotations in evidence of these generalizations: “they [scammers] were able to do it to my client because he has social media, makes a lot of TikToks and Instagram videos. (…) they had his voice” (P3.7); “they [Google] wanted to put it out there so quickly that they’re not even sure of the capabilities of their AI. So it can evolve on its own and teach itself” (P3.6); “They [Delta Airlines] have my face. Trust me. Everyone has your face” (P3.8); “they didn’t tell me that it was a deepfake. They had a fake name and everything, say doctor … whatever name it was, MD, and it was the deepfake made by eight or nine doctors getting one doctor [sic]” (P3.1); “I know they [AI researchers] have a narrow scope [of AI] and the general one. And they don’t have the general one as far as they say, that’s the one that actually teaches itself” (P3.13); “I’m like ‘no’ [to eye scans]. They get you” (P3.7); “TikTok, they use artificial intelligence machine learning behind to give you suggestions that you like” (P3.12).

### Attitude

There is a marked difference in the way the three FGs think and feel about AI/deepfake. The “Engaged public” (FG#1) has a generally positive attitude, marked by confidence in the science/scientists and engineering/engineers, and optimism about the benefits that are likely to flow from ongoing developments in these areas. Some of these attitudes are anchored firmly in the experience of taking the *Make a Fake* class, but others are more generally associated with AI/deepfake itself. Here are some illustrative quotations: “I was really interested in taking this course to learn more about like how these different technologies are being used in the arts in particular” (P1.3); “I hadn’t actually gotten around to doing anything hands on with it [generative AI], and this [workshop] seemed like a good excuse to kind of get up close and personal with these tools” (P1.8); “There’s a whole … like personal art that you can create [with AI]” (P1.7); “I’ve just got a better understanding of how it could be used and how it could be used beneficially, like I said, for designing your garden or designing your pottery before you actually get to it, or creating a vision of something, you know, just for the fun” (P1.9); “I like the look of that generated [deepfake] clone!” (P1.6); and, “I think [deepfake is] playing a really important role of [sic] educating the public that it’s possible to synthesize voices for these public figures” (P1.8).

The “Interested Public” (FG#2) displays attitudes that were generally ambivalent, with a certain negativity directed at some of the way(s) in which this technology might be adopted in society as a whole. It is important to note here that no participant focuses only on the positive aspects of the technology. Again, here are some illustrative quotations from the transcripts: “I refer to my phone and my laptop as my external brains. (…) My cognitive map in my own city that I live in is abysmal, because everyone [relies on?] Google Maps to get everywhere. And I always have it, and it’s not a problem, and it’s the same with any other technology” (P2.1); “in a way, [AI] it’s democratizing art, in the sense that it’s another tool; but on the flip side, you have people that have dedicated their lives to studying music or studying art in general” (P2.9); “To me the saddest part of this AI development, from a personal perspective, is the situation of small research labs … because we cannot compete with monsters [big tech companies]” (P2.6); “there needs to be laws and standards that involve new media, a lot of stuff to do there [AI art]” (P2.14).

Finally, among the “General public” (FG#3) there are many examples that serve to illustrate this group’s generally negative attitude toward AI/deepfake: “[deepfake is] something that people should be protected of [sic]” (P3.3); “I receive so many scam phone calls that nowadays if I don’t know the number, I receive an SMS or a call, I just think it’s a scam. So it’s the same thing with these deepfake videos” (P3.11); “It’s really, really scary in the reality of old people. (…) you’re talking to a lot of people that are not savvy in this world, that have no clue” (P3.5); “How do you know what’s reliable?” (P3.5); “There are doctors, if you go into some of those online tele-health visits, they can be really quickly [sic] just to get an antibiotic. Some of them are deepfakes. And it’s kind of terrifying” (P3.1).

### Critical evaluation and tone

Differences between the three conversations in nuance, in emotionality and in degree of technicality are marked. In FG#1 there is a clear air of optimism throughout. The opinions provided are often nuanced, as a majority of participants made an effort to express both positive and negative aspects of AI/deepfake. Here are some illustrative quotations: “I kind of just want to see where it’s going. I think there are a lot of scary impacts, but I think there are potentially a lot of ways we can grow from it. And I think, ultimately, knowledge has always been something that is advanced and I guess we’ll just see what happens” (P1.2); “[it would be a good thing] teaching the use of the technology alongside the ethical implications that surround it” (P1.3); “Attribution is very important. (…) I think that you need that honesty as to what tools you’re using, what medium you’re working in” (P1.9); “most of the time when I hear about any of this technology, it’s very doom and gloom and scary and alarmist … so I liked hearing that (…) there are just organizations and things starting that seem to be catching up and putting out ways for us to be able to identify the real from the fake. So I thought that was kind of comforting” (P1.12).

Note that the tone in which these opinions are expressed is relatively restrained and unemotional.

Within FG#2 half of the participants presented nuanced opinions while the other half focused on negative aspects—not of the technology itself, but of the ways in which this technology was being adopted by the rest of society. In the quotations that follow, we provide two illustrations of nuanced opinions (purely negative opinions are considered above): “I think that just takes time for the society to get along with AI and for AI to get along with society” (P2.8); and “We can easily tell when there’s a bad Photoshop, but when there’s a good Photoshop, it can be very difficult. So has this been kind of like sitting on the back burner for a lot longer than just us sitting down at this table?” (P2.3). In FG#2 the tone is less restrained, moderately emotional and quite technical. Deepfakes are variously referred to as being “beautiful” (P2.12) and “nefarious” (P2.4). Again, within this group there is extensive use of terms such as “machine learning” (P2.3, P2.12), “Bayesian method” (P2.3), “CG” (P2.1; P2.2) and “Turing test” (P2.1). Here is a further example of the use of quite technical language: “It’s just math behind the scenes [of AI algorithms]. So there’s nothing super special or super inherent, it’s just the data itself” (P2.3).

FG#3 participants expressed the most extreme—and generally negative—positions (a majority of FG#3 gave voice to strongly negative opinions, but just two participants maintained a purely positive position). Here are some illustrative quotations: “How would you be safe from something like that?” (P3.4); “You may do [detect a fake] or believe you do, but I don’t believe you could tell a hundred percent of the time. I don’t believe you could” (P3.5); “there will be a program that will take your voice and it will use that to then scam someone in your family, whether it’s trying to get money or whatever” (P3.7). Again, we note that the tone of FG#3, in keeping a discourse of extremes, is more emotional and less technical. There is extensive use of relatively emotional vocabulary, such as “crazy/craziness” (P3.4; P3.5; P3.8; P3.12), “obscene” (P3.1), “terrifying” (P3.1), “creepy” (P3.8) or “cool” (P3.8). Here is one particularly striking example of the deployment of such vocabulary: “I could say anything I want in your voice. And then post it online and you would never know it wasn’t you. It’s creepy. Yeah, I don’t like it” (P3.8).

### Cultural and media associations

FG#1 does not pay special attention to the media; only three individuals provide some mention of social networks such as “TikTok” (P1.5) and “Twitter” (P1.8) or refer generically to “social networks” and “mainstream media” (P1.7). Cultural images are not abundant among this group either, although some can be traced: “The one [deepfake] where you get an actor that looks a lot like Tom Cruise and you get a voice that sounds a lot like his” (P1.9); “I do know about the one of him [deepfake of Ben Shapiro] playing Minecraft with Biden” (P1.8); “If somebody sees Mother Teresa saying that she went to a nightclub, there might be some suspicion that this isn’t quite right, and they might look a little closer” (P1.5).

FG#2 exhibits a higher, but still moderate presence of terms referring to cultural images, media, and social networks. Such references among this group are only rarely concretized in specific examples (e.g. only one person (P2.11) explicitly refers to TikTok). Thus, we find terms here like “Hollywood” (P2.4), “movies” (P2.2) and “film” (P2.14) used generically to refer to computer-generated imagery (CGI) technology when highlighting the accuracy of AI tools, or the term “comic books” (P2.8) used to express the idea that deepfake is a source of entertainment. Among this group, two participants only make reference to deepfakes of politicians—one mentions Joe Biden, Donald Trump and Barack Obama (P2.12), and the other speaks about “presidents playing video games together” (P2.2). Distinctively, there are several references to governmental and corporate entities, including “DARPA” (P2.3), “Microsoft” (P2.4; P2.6) and “OpenAI” (P2.14; P2.6). Here are two further illustrative quotations: “Can there be good things from it [deepfake]? Like … learning history from George Washington?” (P2.3); and, “My favorite social media reel in the entire world is president Biden and Trump talking about the hardcore punk scene, and then Obama rolls up at the end” (P2.12).

FG#3 exhibits close connections with various social networks, which are mentioned repeatedly throughout the conversation, such as “TikTok” (P3.2; P3.5; P3.7; P3.8; P3.13), “Facebook” (P3.4; P3.5; P3.8) and “YouTube” (P3.12). Noteworthy are the mentions of viral deepfakes circulating in social networks and echoed by the mass media, associated with both political figures such as Joe Biden (P3.1; P3.2; P3.8; P3.13), Donald Trump (P3.1; P3.8; P3.13) and Boris Johnson (P3.2, P3.12) and famous actors such as Tom Cruise and Brad Pitt [P3.8]. In addition, references to movies such as “Indiana Jones” (P3.11) are brought up to illustrate the technical capacity of deepfake when it comes to rejuvenating actors like Harrison Ford (P3.11), but also “I, Robot” (P3.6) and “Jurassic Park” (P3.4), are mentioned in order to express concern about how a technoscientific development can end in social disaster. “La La Land” (P3.5) is referenced in order to condemn the echo-chamber of highly technological subsets of society that discuss these issues without taking into account the reality of non-digitally literate groups. All in all, there is a much larger presence of cultural and media associations here, as can be seen in the following quotations: “there’s one [deepfake] where someone got a deepfake with Joe Biden basically recreating a Nazi speech, like a full 45 minute Nazi speech” (P3.1); and “I thought it was real for a very long time until somebody told me that was not Tom Cruise. And I had to Google it and I [was] shocked” (P3.8).

### Frames

Two frames have been proposed in the academic literature that seem relevant to the FG conversations presented here: “Social Progress” and “Pandora’s Box.” “Social Progress” frames science and technology as tools to improve quality of life and provide solutions, whereas “Pandora’s Box” frames them as sources of unanticipated and uncontrollable problems (Bingaman et al., 2021; Brewer et al., 2022; Nisbet and Scheufele, 2009). Given that there may be links between individuals’ mental frames and their attitudes (Scheufele, 1999), it is reasonable in this context to assume that the presence of the “Social Progress” frame indicates a positive attitude toward AI/deepfake, while the presence of the “Pandora’s Box” frame indicates the opposite (Brewer et al., 2022). Of relevance here also is the fact that these frames are traceable in some mass media and movie representations of science and technology. In the film industry, catastrophic scenarios that present exaggerations about the potential dangers of scientific research have been posited (Broussard, 2018; Broussard et al., 2019; Denia, 2025), and blockbuster movies such as *2001: A Space Odyssey* (1968), *The Terminator* (1984) and *I, Robot* (2004) have instantiated the supposed threat of AI to humanity as a whole (Perkowitz, 2007).

With this as context, it is interesting to observe the dominance of the “Social Progress” frame among the “Engaged Public” (FG#1). Here are some illustrative examples: “I’d like to look at anything that have [sic] to do with technology that will expand my mind” (P1.10); “I feel like there’s lots of paths that I can then go and get further detail from cuz I like to know what to search for” (p1.8); “There’s a whole … like personal art that you can create [with AI]” (P1.7); “I was interested in this because of, you know, what I’ve seen in terms of, you know, image generation and just the capabilities of it” (P1.9); “I’m imagining making a deepfake of my principal lauding the accomplishments of a rival middle school [Laughs] (…) with his permission, of course” (P1.12); “I was kind of aware of some of the image capabilities and generating images [sic] and, you know, but I’ve just got a better understanding of how it could be used and how it could be used beneficially, like I said, for designing your garden or designing your pottery before you actually get to it, or creating a vision of something, you know, just for the fun” (P1.9); “I’m using it currently to generate backgrounds that I put the actual action I draw on top of it [sic], which makes it a lot faster to do!” (p1.6); “I just wanna explore new things in the [AI] field” (P1.2); and “I’ve been interested in ways in which I work with the machine, and this kind of synthetic media has been like another avenue that I wanted to explore” (P1.4).

In sharp contrast, “Pandora’s Box” is an important frame for the “General Public” (FG#3). Among the participants in FG#3, AI/deepfake are widely perceived in terms of threat and loss of control. Within this frame, participants believe that AI/deepfake is advancing faster than their own or society’s abilities to keep up with it, and there is resulting uncertainty and unease about how it will evolve in the future. Here again are some examples: “there’s the fear of losing the human control of what’s going on. Like I think of the movie ‘I, Robot,’ with Will Smith, where it’s like the robots kind of took over and they’re like ‘we need to protect you from yourself,’ kind of thing … it can evolve on its own and teach itself” (P3.6); “In Jurassic Park they were talking about how they weren’t prepared for what this was becoming. ’Cause they were prepared to market it, give it to people, show it to people, but they did not know what it could evolve into” (P3.4); “you have those kids on TikTok, they’re able to literally invent technology. It’s badness. The world’s improving at a rate that it’s not able to keep up with. AI, for a lot of that [sic] cars that are self-driving … still runs [sic] over people. … Over in California, the other day the cops weren’t able to stop it and they had to shoot out the tires. I think it was ’cause it was just gonna keep on driving and just go into a river.” (P3.13); “there will be a program that will take your voice and it will use that to then scam someone in your family, whether it’s trying to get money or whatever” (P3.7); “they [Google] wanted to put it [AI] out there so quickly that they’re not even sure of the capabilities of their AI. So it can evolve on its own and teach itself” (P3.6).

Neither “Social Progress” nor “Pandora’s Box” really apply as meaningful frames for the conversation among the “Interested Public” (FG#2). Here, instead, we detect the presence of a frame that has not, to our knowledge, been previously recognized in the literature on public attitudes to science and technology. We term this frame “Deficit Model,” because of its obvious similarity with an idea that originates in the critical sociological literature on the public understanding of science. There, for some time, it has been observed that many advocates and practitioners of public understanding of science appear to operate with an implicit model, according to which the relationship between scientists and nonscientists is best understood as a relationship between knowledge (scientists) and lack or deficit of knowledge (the public), with the implication that public ignorance is the key driver of the dynamical relationship between the two (e.g. Brossard and Lewenstein, 2010; Sturgis and Allum, 2004). This way of thinking fits well with the ways in which FG#2 participants express their views about the relationship between AI/deepfake and society. Here are some illustrative quotations: “people aren’t willing to take the time to actually learn what goes into making these algorithms work. And that would be the biggest hindrance or concern that I would have” (P2.3); “My mom, my cousins, my friends that I went to college with… like, they do not have the same structural framework that everyone here I’m guessing has, or any visitor to the MIT Museum would have. So how do we make sure that everyone has the same framework to understand and talk about these things?” (P2.12); “it might be more prudent to have a conversation less so about controlling, you know, what they [kids] know about it [AI] and more so building a framework around teaching them how to use it wisely. Because they will almost always be ahead of the curve on that one” (P2.2); “to make people able to sort of think flexibly and critically about these issues [AI/deepfake], it’s kind of, I think, the missing piece maybe” (P2.11).

## 5. Discussion

Although there are some basic similarities between our three FGs, it is clear that their varied social distances from deepfake technology are associated with some systematic differences in the ways in which FG participants thought about this technology. To summarize these differences we offer the following brief caricatures of the three separate focus group conversations.

FG#1 was a conversation among novice deepfake practitioners. As budding practitioners will, the discussants behaved for the most part like insiders: they spoke about the technology in the first person; they spoke about it with a certain amount of confidence; they were alive to many potential benefits, but aware of the potential for misuse by bad actors; and they were also concerned about what others (by implication, outsiders) might think of the technology. But on the whole, this group was positive about the technology’s implications, and concerned more than anything about its own ability to keep up with the rapidity of technological developments in the field. The prevailing frame of reference for this exchange was “Social Progress.”

FG#2 was a conversation among learners who were keen to know more about this and other technologies. As learners sometimes are, they were aspirant insiders, aware of their own—and, especially, others’—shortcomings, and concerned above all about how such shortcomings on the part of others might lead to misapprehensions and misunderstandings that would stand in the way of progress. Theirs was not just talk about technological and social progress, it was also talk about the ways in which alarmist and ill-informed speculation among people less knowledgeable about AI/deepfake than themselves might stand in the way of the potential benefits that this technology might bring. For these reasons, we identify the predominant frame of reference for this conversation as “Deficit Model.”

FG#3 was a conversation among people who, for the most part, felt themselves to be outsiders. Far and away the most negative of our three samples, this group worried a lot about the question: what might “they” do next with a technology over which “they,” and they alone, had any kind of control? And as the group pondered this question, so it conjured multiple, more-or-less familiar and more-or-less negative images from mass media and movie representations—images of politically compromising deepfakes of former UK Prime Minister Boris Johnson, images of mischievous deepfake videos circulating on TikTok, and images of movie representations of technologically inspired disasters, such as *Jurassic Park*. Here, indeed, the predominant frame of reference was what is known in the literature as “Pandora’s Box.”

It will be worth paying more attention to what we have termed “Point of View” than is customary in research on public attitudes to science. Choices made among personal pronouns are revealing of individuals’ and groups’ assumptions about their relationships to the worlds of science and technology. Do individuals perceive themselves as active members of these worlds (first person), aspirant or would-be members of these worlds (third person, with the object of attention being other, less well-informed members of the public), or passive spectators or even victims of these worlds (third person, with the focus on science and technology as remote sources of authority)? Our results here appear to connect with wider sociological literatures on “self-efficacy,” which have emphasized the importance of subjective feelings of competence, effectiveness, and causal agency for many life outcomes (e.g. Gecas, 1989). It will surely be worth further research to determine whether, as we suspect, the very language in which members of the public capture their thoughts and feelings about technological issues is itself revealing of different levels of self-efficacy with respect to science and technology.

Our deployment of framing analysis suggests that members of the public who are at different conceptual distances from science and technology may frame these subjects in quite different ways. “Social Progress” and “Pandora’s Box” are alternate frames that have relatively widespread currency within contemporary American culture, and it is not surprising that they apply to some of the FG conversations described. Hitherto, however, it has been widely assumed that the so-called “Deficit Model” is characteristic of special groups, including many science communicators. The results reported here suggest that this model may apply to a much larger section of the public, namely what we have termed the interested public for science and technology. The implications of this finding for science communicators whose work may be targeted principally at precisely such interested publics are surely worthy of further exploration.

Our FG participants viewed AI/deepfake from three very different conceptual distances, and as they did so they saw three significantly different things. For one thing, those closer to the technologies appear to have had more nuanced assessments of the impact of these technologies on wider society. Also, there is evidence that those closest to AI/deepfake (FG#1) preferred their own personal—and generally positive—experiences over the less personal and generally more negative social images that were available to them in mass media and the movies; whereas those farthest away from AI/deepfake (FG#3), having far fewer personal experiences on which to call, relied much more heavily on the social images of AI with which they were at least generally familiar. Since these social images were prevailingly negative (not least, in our participants’ perception of them), the overall stance that FG#3 participants took toward deepfake and related technologies was correspondingly negative.

We should be careful not to allow these characterizations to pass into judgments—as, for example, that attitudes in FG#1 were more “discerning” or more “rational” than those in FG#3. Given the general levels of social anxiety about the wider ethical and social impacts of Artificial Intelligence in the 2020s, it could as well be argued that the more positive attitudes of FG#1 were overly influenced by narrower personal and professional interests, while the more negative attitudes of FG#3 represented a wider view of larger concerns. The point here is not to judge the various attitudes displayed from some supposedly independent perspective, but rather to understand what these different attitudes may tell us about the relationship between social distance from and attitudes toward technology among different segments of the public.

The general verdict of this study is that in the case of deepfake technology greater social distance did not lend greater enchantment. On the contrary, in the United States in 2023 social distance from AI/deepfake appears to have served to multiply and reinforce various forms of disenchantment. One form of disenchantment was an increasing level of distrust in the motives and intentions of the authors of these technologies. But above all, greater social distance allowed prevailing social images and representations to shape our participants’ responses to a technological area of which they had little firsthand experience, in which they were not substantially invested and over which they felt they had no meaningful control. Conversely, Focus Group #1 comprised people who felt themselves to be participants in deepfake technology, albeit of a rather junior sort; and with this sense of participation came corresponding senses of confidence and optimism about the potential for the constructive use of this technology. In this particular instance, it would appear that to “know” the technology was indeed to “love” it.

If there is a general conclusion to be drawn from this study, it is *not* that social distance lends disenchantment with science and technology; rather, it is that social distance decreases the influence of personal experience and increases the influence of social images and representations in the formation of attitudes to science and technology. This idea is consistent with the finding from survey studies that the relationship between knowledge and attitudes in relation to specific science- and technology-related issues is complex and issue-dependent (see Section 1); also, it is an idea that is open in principle to further empirical exploration. For example, we have dealt here with the relationship between social distance and attitudes in relation to a specific area of science and technology where the social images available to our focus group participants were mainly negative. To put it bluntly, media coverage of AI in the United States in the first half of 2023 was extraordinarily negative—at times, even apocalyptic—and it is not surprising, perhaps, that members of the public for whom these were the chief sources of knowledge would have generally negative attitudes.

What, though, if we were to repeat this study in a very different scientific or technological issue area, where prevailing social images were generally positive? Here, our general conclusion would predict that social distance would indeed lend enchantment, as the influence of prevailingly positive social images and representations increased with increasing social distance from the relevant issue area. This is an empirical study that could in principle be undertaken. However, the challenges facing researchers in such a study should not be underestimated. In order to conduct this kind of study, it will be necessary first to select an appropriate scientific or technological topic. To be suitable for purpose, this topic will need to be: (1) socially salient (so that most members of the public are at least generally familiar with it); (2) socially accessible (so that some members of the public have a much closer relationship to it than others); and (3) the subject of generally positive social images and representations.

Identifying a topic that fulfills all of these criteria will be no easy task. In a previous era in the United States, one or more aspects of space exploration might have been suitable. Today, perhaps some areas of astrophysics—research on black holes, for example, or the search for life elsewhere in the universe—might fit the bill, though there are questions to be asked about quite how socially salient such research really is. Alternatively, we might look for a topic from within the general area of medical science. (Were it not for the fact that it has become increasingly politicized in the United States, an obvious candidate topic here might have been the recent development of COVID-19 vaccines.) We would welcome other candidate topics. Whatever the topic chosen, further research must be timely, in relation to scientific developments and public representations, and well-targeted, in relation to focus group composition. We hope that such research will be undertaken.

## Supplemental Material

sj-docx-1-pus-10.1177_09636625251374850 – Supplemental material for Does “distance lend enchantment”? Public attitudes to deepfake technology in the United States Supplemental material, sj-docx-1-pus-10.1177_09636625251374850 for Does “distance lend enchantment”? Public attitudes to deepfake technology in the United States by Elena Denia and John Durant in Public Understanding of Science

## Notes

1. While the most widely publicized uses of deepfake are deceptive and morally problematic (e.g. to misrepresent political figures, or to insert celebrities into pornographic videos), it is important to remember that there are putatively positive and morally unproblematic uses of this technology—for example, in art, medicine, and film-making. For more details about these positive uses of deepfake, see: https://towardsdatascience.com/positive-use-cases-of-deepfakes-49f510056387.

2. By “distance” in this context, we mean the degree of general familiarity with (knowledgeability about, experience with, etc.) deepfake technology, emphasizing again that none of our chosen publics are themselves deepfake technology experts.
