# Supplementary material for: Does “distance lend enchantment”? Public attitudes to deepfake technology in the United States
Source: Public Underst Sci. 2025 Oct 26;35(2):236–52. doi: 10.1177/09636625251374850 (PMC12852486; doi:10.1177/09636625251374850)
Supplement: sj-docx-1-pus-10.1177_09636625251374850 – Supplemental material for Does “distance lend enchantment”? Public attitudes to deepfake technology in the United States [file sj-docx-1-pus-10.1177_09636625251374850.docx]

**Supplemental material**

Title: Does ‘Distance Lend Enchantment’? Public Attitudes to Deepfake Technology in the United States

Authors: Elena Denia and John Durant

This document contains additional supporting information for the article *“Does ‘Distance Lend Enchantment’? Public Attitudes to Deepfake Technology in the United States”* by Elena Denia and John Durant. It includes:

- **Appendix A**: Background information on participants in the three focus groups, presented in their own words.
- **Appendix B**: Full protocols used for the focus groups, including the sequence of questions and prompts.

These materials are provided to enhance transparency and allow readers to assess the study’s methodology and evidence in greater detail.

##

## Appendix A

In the words of to our participants, their backgrounds are as follows:

Focus Group #1: Journalist [P1.1]; computer science student [P1.2]; art curator and educator [P1.3]; ceramic artist [P1.4]; major in communication [P1.5]; professor of visual culture in new media [P1.6]; social entrepreneur [P1.7]; software engineer [P1.8]; photographer and software engineer [P1.9]; hotel industry [P1.10]; cognitive science [P1.11]; middle school science teacher [P1.12].

Focus Group #2: Illustrator and climate scientist [P2.1]; optical engineer [P2.2]; software engineer [P2.3]; Microsoft [P2.4]; computer scientist [P2.5]; computer scientist (AI and space) [P2.6]; material scientist [P2.7]; space engineer [P2.8]; IT [P2.9]; do not say [P2.10]; surgical robotics [P2.11]; education and digital poetry [P2.12]; teacher [P2.13]; mathematician and digital marketing [P2.14].

Focus Group #3: human development in culinary arts student [P3.1]; do not say [P3.2]; do not say [P3.3]; finance [P3.4]; nurse practitioner [P3.5]; seventh grade math teacher [P3.6]; pharmaceutical [P3.7]; Certified Public Accountant [P3.8]; MIT student [P3.9]; do not say [P3.10]; medical devices company [P3.11]; Banking consultant with machine learning knowledge [P3.12]; wood worker [P3.13].

## Appendix B

**Focus Group #1 Protocol**

INTRODUCTION

We, John Durant (MIT Museum director) and Elena Denia (MIT Postdoctoral Fellow), are conducting this Focus Group to help us better understand how society thinks about deepfake technology. We would like to record the session if you agree, but nothing attributable will be used. We may use the results for our research and related publications, but we will never attribute any particular statement to any particular person. Please give a nod if you consent.

FIRST: You all mentioned this in the first evening (but we want to record it now), could you say very quickly: (i) what your background is, and (ii) why did you want to take the class?

SECOND: How was the class?

Have you been surprised by anything after taking the course?

Did you expect deepfakes to be so easy/difficult to make?

THIRD: Now that you know how to make a deepfake, do you plan to use this ability?

What kind of uses do you imagine?

FOURTH: Is the way you think about this technology different once you have taken the class?

After the course, do you see any particular benefits or harms?

How do you think deepfake will affect the way you and others perceive and consume digital information?

In your opinion, will it significantly increase distrust in online sources of information?

Do you think you are now able to detect a deepfake?

Are you concerned that more and more people are able to make deepfakes?

Should we teach students how to make a deepfake?

FIFTH: You now learned a particular kind of fake which uses Artificial Intelligence (AI), what thoughts do you have about our future as a society with AI?

Are you optimistic/pessimistic and, if so, what are you excited/worried about?

Are you enthusiastic/concerned with the rapid improvement of AI technology?

**Focus Group #2 Protocol**

INTRODUCTION

I am John Durant (MIT Museum Director) and she is Elena Denia (MIT Postdoctoral Fellow). We are conducting this Focus Group to help us better understand how society thinks about AI. We would like to record the session if you agree, but nothing attributable will be used. We may use the results for our research and related publications, but we will never attribute any particular statement to any particular person. Please give a nod if you consent.

FIRST: Could you say very quickly: (i) what your background is, and (ii) to what extent you are interested in science & technology?

SECOND: What thoughts do you have about the development of Artificial Intelligence and our future as a society?

What is AI in your own words?

Are you optimistic/pessimistic and, if so, what are you excited/worried about?

Are you enthusiastic/concerned with the rapid improvement of AI technology?

THIRD: Do you know what a deepfake is?

What comes to mind?

DEFINITION: Deepfakes are digitally manipulated videos that show people saying or doing things they never really did.

FOURTH: Do you see any particular benefits or harms of deepfake technology?

How do you think deepfake will affect the way you and others perceive and consume digital information?

In your opinion, will it significantly increase distrust in online sources of information?

Do you think you are able to detect a deepfake?

Are you concerned that more and more people are able to make deepfakes?

Should we teach people how to make a deepfake?

Is there anything else about deepfakes that you want to tell us?

**Focus Group #3 Protocol**

INTRODUCTION

We are MIT Researchers John Durant (MIT Museum Director) and Elena Denia (Postdoctoral Fellow), conducting this Focus Group to help us better understand how members of the public think about some developments in new technology. (Please don’t worry: you don’t need to know anything special to do this!) We would like to record the session if you agree, but nothing attributable will be used. We may use the results for our research and related publications, but we will never attribute any particular statement to any particular person. Please give a nod if you consent.

FIRST: Could you say very quickly: (i) who you are, and what you do for a living (your occupation)?

SECOND: I wonder if you could say what sorts of things come to mind when you think about new technologies in general?

THIRD: Getting a bit more specific, what comes to mind when you think about Artificial Intelligence?

What is AI in your own words?

Are you optimistic/pessimistic and, if so, what are you excited/worried about?

Are you enthusiastic/concerned with the rapid improvement of AI technology?

FOURTH: Now, we want to explore one aspect of AI: Deepfake technology. Do any of you happen to know what Deepfake is?

What comes to mind?

DEFINITION: Deepfakes are digitally manipulated videos that show people saying or

doing things they never really did.

FIFTH: Do you see any particular benefits or harms of Deepfake technology?

How do you think deepfake will affect the way you and others perceive and consume digital information?

In your opinion, will it significantly increase distrust in online sources of information?

Do you think you are able to detect a deepfake?

Are you concerned that more and more people are able to make deepfakes?

Should we teach people how to make a deepfake?

Is there anything else about deepfakes that you want to tell us?
